# On Warming and Ventilating; with Directions for Making and Using the Thermometer-Stove, or Self-Regulating Fire, &c.

**Published:** 1838-04

**Authors:** 


Art. IV.—*On Warming and Ventilating; with Directions for making and using the Thermometer-Stove, or Self-regulating Fire, and other new Apparatus.* By Neill Arnott, m.d., Physician Extraordinary to the Queen.—*London,* 1838. 8vo. pp. 138.

This is an admirable book; and, although not (strictly speaking) a medical one, it ought to be in the hands of every member of the profession. The warming and ventilating of chambers and houses are so intimately connected with the prevention and cure of diseases, that every physician and surgeon ought to understand them thoroughly; or, at least, the principles on which these most important measures rest: and we think it is not going too far to say that no book, before the publication of Dr. Arnott's, contained the exposition of these principles in such a clear and simple form as rendered the understanding of them easy to every one But, besides the much clearer exposition of what was already known, Dr. Arnott's book contains a great deal of most important information which is entirely new. It is, indeed, like the "Elements of Physics," by the same author, literally full of the most interesting knowledge and the most ingenious contrivances, all planned and directly destined for the improvement of the arts of life, and the amelioration of man's condition as a social and civilized being.

The invention of greatest interest and value is the *thermometer-stove,* named in the title-page; an invention which appears to us calculated to produce a total change in the mode of warming houses, and with a saving of expense in the article of fuel, (to say nothing of its probable benefits in improving health,) which will cause it to be hereafter regarded as of national importance. We will not attempt here to describe the stove: we will only say that it may be manufactured, from Dr. Arnott's description, by any experienced white-smith, at a small expense, and that it has been proved to have the power of keeping up an incessant and unvarying degree of temperature in a room, at the cost in fuel of threepence, or, we believe we may say, three half-pence, in twenty-four hours!

A general expression of the qualities of the thermometer-stove is, that it possesses all the advantages of steam or hot-water warming, with many peculiar to itself. The following are the chief of these advantages:—1, Economy of fuel; 2, uniformity of temperature; 3, perpetual operation; 4, no smoke; 5, no dust; 6, no danger to persons or property; 7, obedience to command; 8, trifling original expense; 9, little trouble attending it; 10, moveable from room to room; 11, graceful form; 12, no sweeping boys, &c. Of all these advantages, No. 3—its perpetual operation, or being always alight,—is that which captivates us most; perhaps, because it comes most home to our business and bosom.

"Its importance is perceived by reflecting on the dependence of persons generally, in winter, on the regularity of servants or others, by being obliged, in the mornings, to wait in bed, until fires are lighted and rooms are put in order. The early riser, student, or man of business, with his ever-burning stove and alarum clock, is independent of the seasons. In England, many persons acquire the habit of lying abed in winter, as above explained, and then continue it in summer, and become eventually sluggards through life, from the accident of our imperfect domestic economy." (P. 49.)

We cannot conclude without expressing our sense of the almost unexampled liberality of Dr. Arnott, in refusing to take out a patent for this admirable invention; a liberality which has only one parallel, which he himself afforded when he made the same sacrifice in the case of his other transcendent gift to afflicted humanity, the water-bed. The extent of the sacrifice in the present case appears to us literally immense; as we cannot but believe that the thermometer-stove will be used by thousands and tens of thousands, if, indeed, it do not eventually become an article of universal use. It is in instances like these that we are proud to see evidence that that combination of science, dignity, and humanity, which we would fain regard as inherent in the true medical character, still lives among us, to put to shame the ignorance, and quackery, and selfishness which we see too often striving to degrade it.

